# Trends in socioeconomic inequalities in smoking prevalence, consumption, initiation, and cessation between 2001 and 2008 in the Netherlands. Findings from a national population survey

**DOI:** 10.1186/1471-2458-12-303

**Published:** 2012-05-18

**Authors:** Gera E Nagelhout, Dianne de Korte-de Boer, Anton E Kunst, Regina M van der Meer, Hein de Vries, Boukje M van Gelder, Marc C Willemsen

**Affiliations:** 1 STIVORO Dutch Expert Centre on Tobacco Control, PO Box 16070, 2500 BB, The Hague, the Netherlands; 2 Maastricht University (CAPHRI), Maastricht, the Netherlands; 3 Academic Medical Centre, University of Amsterdam, Amsterdam, the Netherlands; 4 National Institute for Public Health and the Environment (RIVM), Bilthoven, the Netherlands

**Keywords:** Netherlands, Smoking, Social class, Trends

## Abstract

**Background:**

Widening of socioeconomic status (SES) inequalities in smoking prevalence has occurred in several Western countries from the mid 1970’s onwards. However, little is known about a widening of SES inequalities in smoking consumption, initiation and cessation.

**Methods:**

Repeated cross-sectional population surveys from 2001 to 2008 (n ≈ 18,000 per year) were used to examine changes in smoking prevalence, smoking consumption (number of cigarettes per day), initiation ratios (ratio of ever smokers to all respondents), and quit ratios (ratio of former smokers to ever smokers) in the Netherlands. Education level and income level were used as indicators of SES and results were reported separately for men and women.

**Results:**

Lower educated respondents were significantly more likely to be smokers, smoked more cigarettes per day, had higher initiation ratios, and had lower quit ratios than higher educated respondents. Income inequalities were smaller than educational inequalities and were not all significant, but were in the same direction as educational inequalities. Among women, educational inequalities widened significantly between 2001 and 2008 for smoking prevalence, smoking initiation, and smoking cessation. Among low educated women, smoking prevalence remained stable between 2001 and 2008 because both the initiation and quit ratio increased significantly. Among moderate and high educated women, smoking prevalence decreased significantly because initiation ratios remained constant, while quit ratios increased significantly. Among men, educational inequalities widened significantly between 2001 and 2008 for smoking consumption only.

**Conclusions:**

While inequalities in smoking prevalence were stable among Dutch men, they increased among women, due to widening inequalities in both smoking cessation and initiation. Both components should be addressed in equity-oriented tobacco control policies.

## Background

Nowadays, mortality rates tend to be higher among lower socioeconomic status (SES) groups in most Western countries [1-3]. The higher prevalence of smoking in individuals from lower SES groups is the single most important cause of socioeconomic differences in mortality [4,5]. The smoking epidemic model describes the trends of smoking prevalence and smoking-attributable mortality in populations over time [6]. In the first stage of the smoking epidemic, smoking prevalence and smoking-attributable mortality is low and rising. In the second stage, smoking prevalence among men rises rapidly, while smoking among women lags behind. Smoking prevalence rates are similar among different SES groups or may be higher among high SES groups. In the third stage, male prevalence declines and female prevalence remains stable. There is a rapid rise in smoking-attributable mortality among men. In the fourth stage of the smoking epidemic, smoking prevalence declines for both men and women and there is a rapid rise in smoking-attributable mortality among women. Smoking prevalence is higher among lower SES groups in this stage and a widening of SES inequalities in smoking prevalence may occur. A widening of SES inequalities in smoking has been found for several Western countries from the mid 1970’s onwards, for example in most U.S. states [7,8], Canada [9], Australia [10,11], New Zealand [12], and most European countries [13-16].

Studies that examined trends in SES differences in smoking focused mostly on differences in smoking prevalence [17], i.e. the proportion of smokers in a population. These studies provide no information about how SES differences in smoking prevalence originate, because the smoking prevalence in a population is determined both by smoking initiation and by smoking cessation. The few studies that have examined trends in SES inequalities in both smoking initiation and cessation found mixed results [8,18]. One study found that widening SES inequalities in cessation are mostly responsible for widening SES differences in smoking prevalence [8], while the other study found that inequalities in initiation are a more important explanation [18]. Knowledge about trends in SES inequalities in initiation and cessation can potentially help to better inform future tobacco control interventions [19]. Furthermore, it is important to examine SES differences in smoking consumption levels, as consumption levels have been found to relate in a dose–response manner to the risks and severity of many smoking related diseases [20]. Therefore, information about trends in SES differences in smoking consumption, smoking initiation, and smoking cessation is required [17].

In the current study, trends in SES differences in four smoking related outcomes are examined for the Netherlands for the period 2001 to 2008. In this period, several tobacco control policies were implemented that could have influenced SES differences in smoking. In 2002, text warning labels for cigarette packages were implemented and a tobacco advertising ban was implemented [21]. In 2003, a youth access law was implemented [21]. In 2004, smoke-free workplace legislation was implemented, which was extended in 2008 so as to include the hospitality industry [22]. Tax increases were implemented in 2001, 2004, and 2008 and intensive national mass media smoking cessation campaigns ran in 2003, 2004, and 2008 [22].

We aim to answer the following research questions: 1) Were there SES differences in smoking prevalence, smoking consumption, smoking initiation, and smoking cessation in the Netherlands in 2001 and 2008? and 2) Did these SES differences change in the period 2001 to 2008? These research questions are answered for both men and women.

## Methods

### Sample

Data were obtained from the Dutch Continuous Survey of Smoking Habits (DCSSH). This is a cross-sectional population survey of respondents aged 15 years and older that is used to monitor smoking habits of the Dutch population, using weekly measurements. The DCSSH is conducted by market research company TNS NIPO for the Dutch expert centre on tobacco control (STIVORO). Respondents for the DCSSH were selected from TNS NIPObase, a database containing more than 140,000 potential respondents who participate in internet-based research on a regular basis. TNS NIPObase panel members are recruited actively by TNS NIPO using telephone and mail.

For the present study, DCSSH data from 2001 until 2008 were used. The questionnaire was sent to a stratified random sample of potential respondents by e-mail. Respondents completed the questionnaire at their own computer using software from TNS NIPO. Approximately 18,000 respondents participated in the survey each year, totaling 144,733 respondents in the period 2001 to 2008. The respondents were representative of the Dutch population of 15 years and older after applying weights for gender, age, education level, working hours, geographic region, urbanization, and household size. Response data were weighted on the basis of stratum weights. Each respondent was provided a weight by TNS NIPO with DIANA software, using an iterative process until there was little deviation between the weighted data and the target strata.

The Central Committee on Research Involving Human Subjects in the Netherlands requires no ethical approval for non-medical survey research.

### Questionnaire

The DCSSH questionnaire assessed demographic characteristics, smoking behaviour and use of smoking cessation aids and contained sections on tobacco control policies and campaigns. For the current study, we used questions about SES indicators, smoking status, smoking consumption, age, and gender.

Although education level is seen as a key indicator of SES [17,23], it has been recommended to include other indicators as well to account for other dimensions of SES that have shown to be independently related to smoking [17]. We, therefore, used both education level and income level as indicators of SES. We categorized education level into three groups: low (primary education and lower pre-vocational secondary education), moderate (middle pre-vocational secondary education and secondary vocational education) and high (senior general secondary education, (pre-)university education and higher professional education). Gross yearly household income level was also categorized into three equal sized groups: low (less than 28,500 Euro = < 25,600 GBP), moderate (between 28,500 and 45,000 Euro = 25,600 - 40,430 GBP), and high (more than 45,000 Euro = > 40,430 GBP).

In line with earlier studies [22,24], current smoking status was assessed by asking ‘do you (ever) smoke or do you not smoke at all?’. Respondents who answered that they smoke were defined as current smokers. Respondents who answered that they do not smoke were asked: ‘have you smoked in the past?’. Respondents who answered that they had smoked in the past were defined as former smokers and respondents who answered that they had not smoked in the past were defined as never smokers. Smoking prevalence was defined as the proportion of all respondents who were current smokers (current smokers/all respondents * 100). In the regression analyses, smoking prevalence was treated as a binary outcome.

Following recommendations from Schaap and Kunst [17], smoking initiation and cessation were examined by calculating initiation ratios and quit ratios. Initiation ratio was defined as the ratio of all respondents who were ever smokers (current + former smokers/all respondents). Quit ratio was defined as the ratio of ever smokers who were former smokers (former smokers/current + former smokers). In the regression analyses, initiation and quit ratios were treated as binary outcomes.

Smoking consumption was measured by asking current smokers how many cigarettes (factory made and/or roll-your-own) they smoked on average per day. In the regression analyses, smoking consumption was treated as a continuous outcome.

Furthermore, age and gender were assessed. Age was categorized into four groups: 15–24 years, 25–39 years, 40–54 years, and 55 years and older.

### Analyses

SES differences in smoking related outcomes (research question 1) were examined by logistic regression analyses (for smoking prevalence, initiation, and cessation) and linear regression analyses (for smoking consumption). Dependent variables were smoking prevalence, smoking consumption, smoking initiation, and smoking cessation. Independent variables were education level and income level (in separate analyses). Regression analyses were controlled for age group and reported separately for men and women in 2001 and 2008. Respondents who did not report their education level (n = 665, 0.5%) were excluded from the analyses with education level. Respondents who did not report their income level (n = 30,661, 21.2%) were excluded from the analyses with income level. Respondents who did not report their income level were significantly more likely to have a low education level (*χ*^2^ (2) = 413.08, p < 0.001), to be younger (F (1) = 238.40, p < 0.001), and to be a current smoker (*χ*^2^ (1) = 61.52, p < 0.001).

SES differences in trends in smoking related outcomes (research question 2) were examined with the regression analyses described above for the survey years 2001 and 2008 taken together and with a dichotomous ‘trends’ variable (0 for 2001 and 1 for 2008) and the interaction between ‘trends’ and education/income level (separate analyses per SES indicator) as independent variables. Regression analyses were controlled for age group and reported separately for men and women. Education level and income level were treated as continuous variables in the interaction analyses so as to limit the number of interaction terms. In all other analyses, they were treated as categorical variables with high education level and income level being the reference categories. Significant interactions between ‘trends’ and education level or income level indicated that the trends in smoking related outcomes differed for people with low, moderate, and high SES.

## Results

### Demographic characteristics

Table 1 shows demographic characteristics of respondents per year. There were no significant differences in education level, gender, and age group between years. However, income level differed significantly between years (*χ*^2^ (14) = 669.19, p < 0.001), which was due to an increase in respondents with higher incomes over time.

**Table 1:** Demographic characteristics from 2001 to 2008 (weighted data)

	2001 n = 18,361	2002 n = 18,212	2003 n = 19,086	2004 n = 18,342	2005 n = 19,344	2006 n = 18,031	2007 n = 14,730	2008 n = 18,627
Education level (%)								
Low	44.4	44.5	44.4	44.5	44.4	44.5	44.5	44.5
Moderate	33.6	33.6	33.6	33.6	33.6	33.6	33.6	33.6
High	22.0	22.0	22.0	21.9	21.9	21.9	21.9	21.9
Income level (%)								
Low	43.6	38.4	34.8	35.7	36.0	35.7	36.2	34.6
Moderate	34.8	37.1	34.2	35.1	34.7	37.3	35.5	36.3
High	21.5	24.6	31.0	29.2	29.2	27.0	28.3	29.2
Gender (%)								
Men	49.1	49.1	49.1	49.2	49.1	49.1	49.1	49.0
Women	50.9	50.9	50.9	50.8	50.9	50.9	50.9	51.0
Age group (%)								
15–24	14.7	14.7	14.7	14.7	14.7	14.7	14.7	14.7
25–39	29.9	30.3	30.3	30.6	30.3	30.3	30.7	30.8
40–54	26.9	26.5	26.5	26.3	26.4	26.5	26.1	26.0
55 and older	28.4	28.6	28.5	28.4	28.6	28.5	28.5	28.6

### SES inequalities

**Figure 1:**
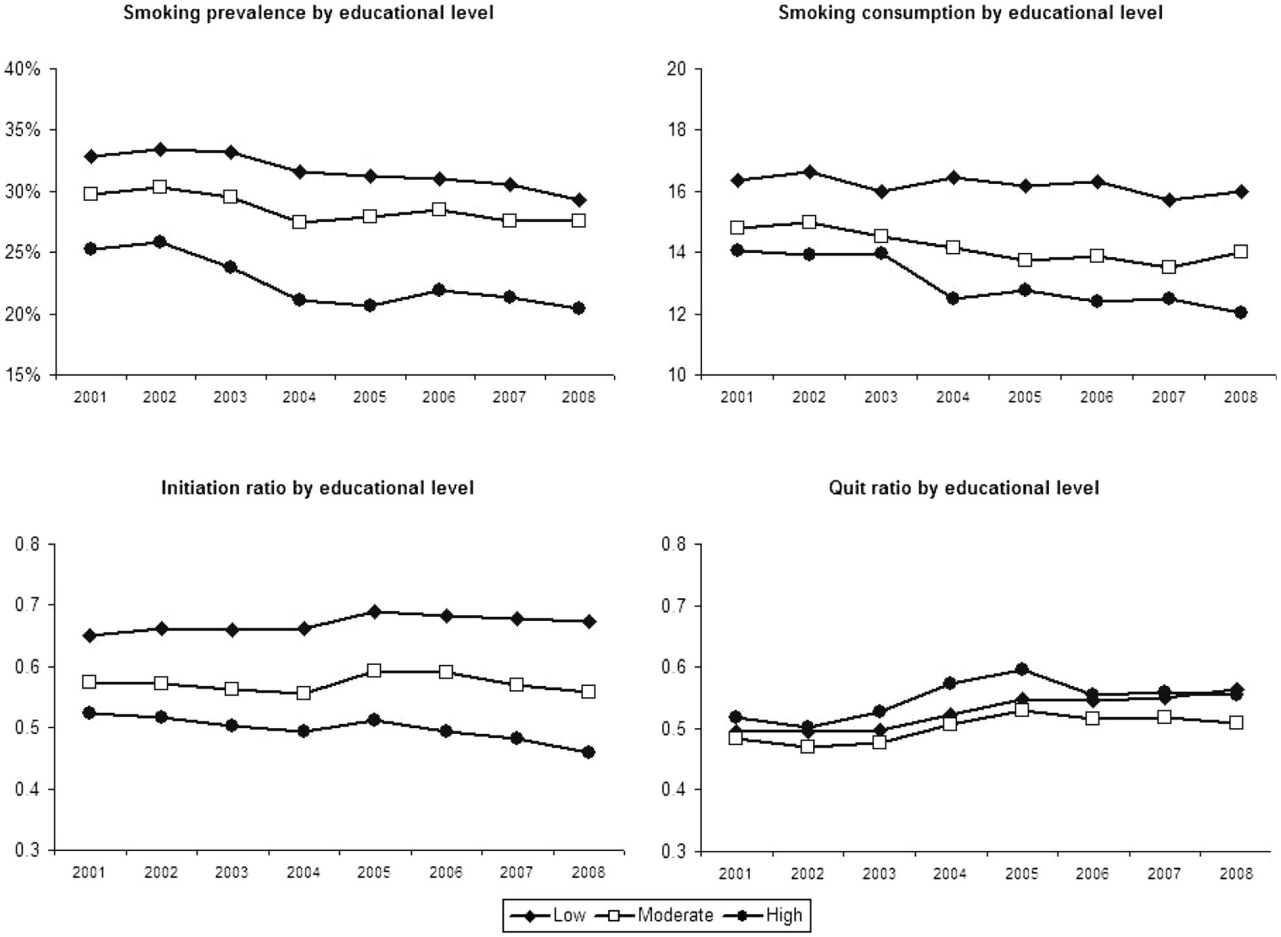
Smoking characteristics of respondents by education level from 2001 to 2008 (weighted data).

**Figure 2:**
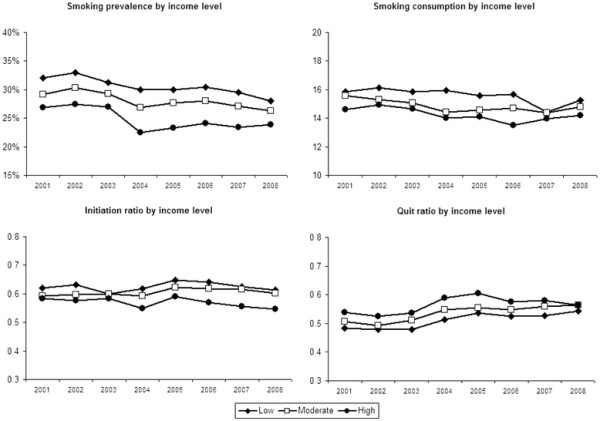
Smoking characteristics of respondents by income level from 2001 to 2008 (weighted data).

Figure 1 shows education level differences and Figure 2 income level differences in smoking prevalence, consumption, initiation ratio, and quit ratio from 2001 to 2008. The figures show that smoking prevalence was higher among respondents with lower education (29% in 2008) and income levels (28%) than among respondents with higher education (20%) and income levels (24%). The regression analyses shown in Table 2 confirmed that the difference in smoking prevalence between respondents with higher and lower education and income was significant among both men and women in 2001 and 2008.

**Table 2:** **Regression analyses**† **in which education level and income level predicted four smoking related outcomes**

	**Men**		**Women**	
	2001	2008	2001	2008
**Smoking prevalence**				
Education level*				
Low	1.75 (1.55 to 1.97)	1.84 (1.62 to 2.09)	1.79 (1.56 to 2.06)	2.26 (1.96 to 2.62)
Moderate	1.32 (1.17 to 1.49)	1.44 (1.27 to 1.64)	1.25 (1.09 to 1.44)	1.62 (1.41 to 1.87)
High	1.00	1.00	1.00	1.00
Income level				
Low	1.33 (1.17 to 1.51)	1.49 (1.31 to 1.70)	1.52 (1.33 to 1.74	1.83 (1.58 to 2.10)
Moderate	*1.13 (0.99 to 1.28)*	*1.11 (0.98 to 1.25)*	*1.12 (0.97 to 1.30)*	1.39 (1.20 to 1.60)
High	1.00	1.00	1.00	1.00
**Smoking consumption**				
Education level				
Low	0.11 (0.06 to 0.17)	0.17 (0.11 to 0.23)	0.11 (0.05 to 0.17)	0.20 (0.13 to 0.26)
Moderate	0.07 (0.02 to 0.13)	0.07 (0.01 to 0.13)	*0.01 (−0.05 to 0.07)*	0.12 (0.06 to 0.18)
High	0.00	0.00	0.00	0.00
Income level				
Low	*0.06 (0.00 to 0.11)*	*0.00 (−0.06 to 0.06)*	0.12 (0.06 to 0.17)	0.15 (0.08 to 0.20)
Moderate	*0.05 (−0.01 to 0.11)*	*−0.01 (−0.06 to 0.04)*	0.06 (0.00 to 0.12)	0.08 (0.02 to 0.14)
High	0.00	0.00	0.00	0.00
**Initiation ratio**				
Education level				
Low	1.87 (1.66 to 2.10)	2.24 (1.99 (2.52)	1.41 (1.25 to 1.59)	1.73 (1.53 to 1.95)
Moderate	1.43 (1.27 to 1.61)	1.47 (1.30 to 1.65)	1.19 (1.05 to 1.34)	1.45 (1.29 to 1.63)
High	1.00	1.00	1.00	1.00
Income level				
Low	1.32 (1.16 to 1.51)	1.51 (1.32 to 1.73)	1.25 (1.11 to 1.41)	1.32 (1.17 to 1.49)
Moderate	*1.14 (1.00 to 1.30)*	1.27 (1.13 to 1.43)	*1.01 (0.89 to 1.14)*	1.21 (1.08 to 1.37)
High	1.00	1.00	1.00	1.00
**Quit ratio**				
Education level				
Low	0.81 (0.70 to 0.94)	0.84 (0.71 to 0.98)	0.69 (0.58 to 0.82)	0.56 (0.47 to 0.67)
Moderate	*0.95 (0.81 to 1.11)*	0.83 (0.70 to 0.98)	*0.92 (0.77 to 1.10)*	0.73 (0.61 to 0.87)
High	1.00	1.00	1.00	1.00
Income level				
Low	*0.86 (0.74 to 1.01)*	0.76 (0.64 to 0.89)	0.72 (0.61 to 0.85)	0.56 (0.47 to 0.66)
Moderate	*0.96 (0.82 to 1.13)*	*1.05 (0.91 to 1.22)*	*0.88 (0.74 to 1.05)*	0.78 (0.66 to 0.92)
High	1.00	1.00	1.00	1.00

† Odds Ratios and 95% confidence intervals are given for smoking prevalence, initiation ratio, and quit ratio, Bètas and 95% confidence intervals for smoking consumption. All regression coefficients were adjusted for age group. Regression coefficients in *italics* were non-significant.

* Education level and income level were entered as independent variables in separate analyses.

Smoking consumption levels were significantly higher among lower educated respondents than among higher educated respondents among both men and women in 2001 and 2008. Differences in smoking consumption between respondents with higher and lower income were smaller and only significant among women in 2001 and 2008.

The difference in initiation ratios between respondents with higher and lower education and income was significant among both men and women in 2001 and 2008, indicating that lower SES respondents more often started smoking than higher SES respondents.

Finally, quit ratios were significantly higher among respondents with higher education and income than among respondents with lower education and income among both men and women in 2001 and 2008, with the exception of differences between male respondents with higher and lower income levels in 2001.

### Changes in SES inequalities

Among women, educational differences changed significantly between 2001 and 2008 for smoking prevalence, smoking initiation, and smoking cessation (Table 3). Regression analyses stratified by education level showed that smoking prevalence did not change between 2001 and 2008 among female respondents with low education (Odds Ratio (OR) = 0.95, 95% confidence interval (CI) = 0.86 to 1.04), while smoking prevalence decreased among female respondents with moderate (OR = 0.89, 95% CI = 0.79 to 0.99) and high education (OR = 0.68, 95% CI = 0.58 to 0.80). Initiation ratios increased significantly among female respondents with low education (OR = 1.19, 95% CI = 1.09 to 1.30), while remaining constant among female respondents with moderate (OR = 1.04, 95% CI = 0.94 to 1.15) and high education (OR = 0.88, 95% CI = 0.77 to 1.01). Quit ratios increased significantly among female respondents in all education levels, but the increase was larger among female respondents with high education (OR = 1.60, 95% CI = 1.30 to 1.97) than moderate (OR = 1.21, 95% CI = 1.05 to 1.40) and low education (OR = 1.16, 95% CI = 1.03 to 1.30).

**Table 3:** **Interactions**† **of trends by education level and income level on four smoking related outcomes**

	Men	Women
**Smoking prevalence**		
Trends * education level	*0.99 (0.91 to 1.07)*	0.86 (0.79 to 0.94)
Trends * income level	*0.95 (0.87 to 1.04)*	*0.94 (0.86 to 1.04)*
**Smoking consumption**		
Trends * education level	−0.08 (−0.17 to −0.01)	*−0.04 (−0.13 to 0.04)*
Trends * income level	*0.05 (−0.04 to 0.14)*	*−0.02 (−0.11 to 0.07)*
**Initiation ratio**		
Trends * education level	*0.94 (0.87 to 1.02)*	0.86 (0.79 to 0.92)
Trends * income level	*0.92 (0.84 to 1.01)*	*0.97 (0.90 to 1.06)*
**Quit ratio**		
Trends * education level	*0.99 (0.90 to 1.10)*	1.14 (1.02 to 1.28)
Trends * income level	*1.05 (0.94 to 1.18)*	*1.11 (0.99 to 1.25)*

† Odds Ratios and 95% confidence intervals are given for smoking prevalence, initiation ratio, and quit ratio, Bètas and 95% confidence intervals are given for smoking consumption. All regression coefficients were adjusted for age group. Regression coefficients in *italics* were non-significant.

* Education level and income level were entered as independent variables in separate analyses.

Among men, educational inequalities widened significantly between 2001 and 2008 for smoking consumption and not for smoking prevalence, initiation, and cessation (Table 3). The mean number of cigarettes per day did not change between 2001 and 2008 among male smokers with low education (β = −0.03, 95% CI = −0.07 to 0.01), while the mean number of cigarettes per day decreased among male smokers with moderate (β = −0.10, 95% CI = −0.15 to −0.06) and high education (β = −0.08, 95% CI = −0.16 to −0.01).

Among both women and men, income differences in smoking related outcomes did not change significantly between 2001 and 2008.

## Discussion

We examined changes in socioeconomic inequalities in smoking prevalence, smoking consumption, smoking initiation, and smoking cessation between 2001 and 2008 in the Netherlands. In line with previous studies [7-16], we found that respondents with lower education and income were more likely to be smokers than respondents with higher education and income in 2001 and 2008. Socioeconomic inequalities in smoking consumption, smoking initiation, and smoking cessation are less well documented than inequalities in smoking prevalence [17]. We found that respondents with lower education smoked more cigarettes per day, had higher initiation ratios, and had lower quit ratios than respondents with higher education in 2001 and 2008. Therefore, it is important that tobacco control programs focus on decreasing smoking initiation and consumption and increasing smoking cessation among the lower educated. It seems that focusing only on either initiation or cessation is insufficient.

In this study, we found evidence that SES inequalities in smoking widened between 2001 and 2008 in the Netherlands, but this widening was not the same for women and men and it depended on which SES and smoking indicator was used. Among women, educational inequalities widened significantly for smoking prevalence, smoking initiation, and smoking cessation. Among men, educational inequalities widened significantly for smoking consumption. The widening pattern in smoking, initiation, and quitting among low educated women is especially worrying. In this group, smoking prevalence remained stable between 2001 and 2008 because both the initiation and quit ratio increased slightly. Among moderate and high educated women smoking prevalence decreased because initiation ratios remained constant, while quit ratios increased considerably. Other studies have also found that in countries in later stages of the smoking epidemic, SES inequalities in smoking prevalence are stable among men, while they are widening among women [10,13]. Also, it was found earlier that SES inequalities in smoking consumption are stable among women and widening among men [13].

According to the literature, advertising bans, smoking bans in workplaces, reimbursement of smoking cessation therapies, and increased tobacco taxes have the potential to reduce SES differences in smoking [25]. During our study period, smoke-free workplace legislation (2004), smoke-free hospitality industry legislation (2008), and tobacco tax increases (2001, 2004, and 2008) were implemented in the Netherlands. From our data, it looks like there was a change in SES inequalities in smoking prevalence and consumption between 2003 and 2004, when the smoke-free workplace legislation was implemented together with a tax increase. However, the SES inequalities seem to be widening instead of narrowing. An earlier study also showed that the workplace smoking ban in the Netherlands has increased SES differences in smoking cessation [22]. An explanation could be that the smoke-free legislation was not comprehensive: the hospitality industry was exempted (until 2008) and designated smoking rooms were allowed.

Our study had some important strengths. We used very large, representative samples of the Dutch population over a period of eight years. Therefore, changes in SES differences over time could be detected and generalized to the Dutch population. Also, we examined both education level and income level differences in four smoking related outcomes, including smoking initiation and cessation for both men and women. Our study thus provides a more detailed picture of SES differences in smoking than most studies. This study also had some limitations. First of all, we relied on self reported smoking status. Since there can be differences in underreporting of smoking between SES groups, this could have influenced our results. Second, income levels were unknown for 21% of respondents. These respondents were excluded from the analyses and this may have introduced selection bias. However, since we also used another SES indicator (education level) with only a few missing values resulting in comparable findings, we expect that the missing income data did not bias our results. Furthermore, we used initiation and quit ratios to estimate population level smoking initiation and cessation. These ratios may not be sensitive enough to reflect short term changes in smoking initiation and cessation. However, initiation and quit ratios are suitable for understanding long term population trends [17].

## Conclusion

This study exemplifies that inequalities in smoking prevalence among women and smoking consumption among men may widen in the fourth stage of the smoking epidemic. Although our study provides some insight in trends in SES differences in smoking in populations, more research is needed to find ways to successfully address these differences. While inequalities in smoking prevalence were stable among Dutch men, they increased among women, due to widening inequalities in both smoking cessation and initiation. Therefore, both components should be addressed in equity-oriented tobacco control policies.

## Abbreviations

SES = Socioeconomic status; DCSSH = Dutch Continuous Survey of Smoking Habits.

## Competing interests

The authors declare that they have no competing interests.

## Authors’ contributions

GN conducted the statistical analyses and wrote the manuscript. All other authors advised on the writing of the manuscript. GN, DdK, RvdM, TZ, BvG, and MW were involved in a Dutch report of which this paper is an adaptation. AK and HdV advised on the design of the study and the statistical analyses. All authors read and approved the final manuscript.

## Pre-publication history

The pre-publication history for this paper can be accessed here:

http://www.biomedcentral.com/1471-2458/12/303/prepub
